# Designing Better Resources: Consumer Experiences, Priorities and Preferences Regarding Contemporary Nutrition Education Materials

**DOI:** 10.1111/jhn.70041

**Published:** 2025-03-25

**Authors:** Kelly Lambert, Sophie Bernes, Nicole Buxton, Nisa Gogebakan, Grace Taylor Hennen, Georgia Flynn Caswell

**Affiliations:** ^1^ School of Medical, Indigenous and Health Sciences University of Wollongong Wollongong New South Wales Australia; ^2^ Health Innovations University of Wollongong Wollongong New South Wales Australia; ^3^ Kidney Lifestyle Research Group University of Wollongong Wollongong New South Wales Australia; ^4^ John Hunter Hospital Hunter New England Local Health District Newcastle New South Wales Australia

**Keywords:** consumer preferences, diet sheet, focus group, framework, health education, nutrition education, patient education handout, qualitative research

## Abstract

**Introduction:**

Nutrition education materials are frequently used by dietitians to support counselling and education. Few studies have explored consumer perspectives regarding these resources and none in a contemporary setting post pandemic.

**Methods:**

Purposive sampling was used to recruit a range of Australian consumers to participate in seven focus groups (conducted between April 2022 and May 2024). Each group involved 6–10 participants. Transcripts were inductively coded and thematic analysis was used to identify recurrent themes that best reflected consumer experiences, priorities and preferences regarding contemporary consumer nutrition education materials. Latent and manifest analysis was conducted on annotations made by consumers on consumer nutrition education materials.

**Results:**

Consumers (*n* = 45) articulated four recurring themes: barriers to use (overwhelming volume of information, unclear purpose, credibility), desirable language (plain language, positive messaging), attention to content (minimal key messages, individualised and actionable materials, culturally applicable) and optimal layout and design (appealing and thoughtful visuals, signposting and flow, colour). A framework for the evaluation and development of nutrition education materials was developed based on consumer insights and relevant literature. This framework can be used to improve the quality of future education materials used to support nutrition counselling and education activities.

**Conclusion:**

The findings from this study provide dietitians with practical guidance to design nutrition education materials that meet consumer needs and expectations.

## Summary

- Consumers have clear preferences regarding the design of nutrition education materials.
- Current nutrition education materials possess many features that act as barriers to use including an overwhelming volume of information, unclear purpose and low credibility.
- Features such as plain language, positive messaging, minimal key messages, and individualised, culturally applicable, actionable materials are desired.
- A checklist for the design and evaluation of nutrition education materials has been developed as a result of these findings.

## 1 Introduction

Nutrition education is a formal process of instruction with a client or patient to impart knowledge, provide skills or facilitate behaviour change to voluntarily improve or maintain health [1]. This partnership between the individual and dietitian is central to the provision of client centred care [2]. Strong evidence exists that nutrition education provided by a dietitian is effective and can improve diabetes control [3], dyslipidemia [4], hypertension risk [5], gestational weight gain [6] and diet quality [7].

During the nutrition education process, it is common practice for dietitians to engage in verbal dialogue supported with written nutrition education materials or ‘diet sheets’. This practice is critical given the substantial evidence that recall of key concepts after verbal healthcare interactions is poor [8, 9, 10]. Advancements in our understanding of how people learn and use health information [11] also suggest that written nutrition education materials are used to refresh a person's memory post dietetic consultation [12], provide additional information to increase knowledge to self‐manage their condition [11, 13], prevent misinformation and recall bias [11, 14, 15] and enable the provision of standardised information with which consumers can make health decisions [16].

Surprisingly, there is limited evidence for dietitians regarding the design of written nutrition education materials and the end user experience despite the importance of them in the nutrition education process. The small evidence base on the quality of nutrition education materials is mostly quantitative in nature and evaluates the understandability, actionability, readability and suitability [10, 17, 18, 19, 20, 21, 22, 23, 24] of these materials. In these studies, it has been identified that nutrition education materials are frequently written at a level that exceeds the literacy skills of the general population and recommends visual aids be incorporated into the design [9, 18, 25]. Guidance regarding the development of quality materials is also sparse, with a distinct absence of the consumer perspective. Few studies exist that have explored the perspectives of consumers regarding nutrition education materials and many that have been published are more than 30 years old [26, 27]. A study in 1989 of 37 female consumers from North America identified that features such as colour, clear explanations and features to help personalise content were important [26]. Similarly, work from two studies with consumers on low cholesterol information highlighted a desire for practical skill‐based information [28] and described how contradictory dietary messaging between different resources created confusion and resulted in non‐adherence to advice [29].

Given the important global shift to partner with consumers to provide quality healthcare [30], it is critical to understand the perspectives of consumers regarding written dietary education materials. Therefore, the aim of this study was to explore consumer experiences, priorities and preferences regarding contemporary nutrition education materials and offer guidance for dietitians in clinical practice.

## 2 Materials and Methods

The Consolidated Criteria for Reporting Qualitative studies (COREQ) [31] is used to report the findings of this focus group study.

### 2.1 Participant Selection

Individuals aged 18 years or older with adequate English to participate in focus group discussions were eligible to participate in this study. Participants were recruited from dietetic clinics of one local health district (Illawarra Shoalhaven Local Health District) as well as community groups, professional networks and media advertisement. We purposively sampled a diverse range of participants with a range of clinical backgrounds (heart failure, diabetes, kidney disease, geriatric rehabilitation, stroke, gastrointestinal disorders, healthy general population) as well as demographic characteristics (age, education level, gender, ethnicity, socioeconomic status and experiences with dietetic care). Written consent was obtained from all participants. Reimbursement ($50 Australian dollars) was provided to cover travel costs for attending the focus groups). Ethics approval was obtained from the NSW Health/Illawarra Shoalhaven Local Health District Human Research Ethics Committee.

### 2.2 Data Collection

Seven focus groups were conducted between April 2022 and May 2024 until data saturation was achieved. Focus group discussions were guided by a semistructured interview guide (Table 1). In addition to discussions, participants were invited to provide annotated feedback on a range of contemporary nutrition education materials (*n* = 12, Table 2 and Supporting Information S1: Tables 1 and 2). These materials were described as frequently used materials by local dietitians involved in the study. Four resources were shown in both black and white and colour versions for comment. In addition, one infographic style material was included based on extensive discussion on social media by health professionals when released (British Dietetic Association resource ‘*Eating a plant‐based diet with Chronic Kidney Disease*’) [32]. Readability scores for these materials ranged from Grade 3 to 10 (Table 2). Participants were asked to use red and green coloured pens to make annotations that reflected their preferences or perspectives. Prompt questions surrounding their annotations to materials stimulated further discussions. Think‐aloud techniques [33] were utilised to complement the qualitative approach, avoid interview bias and elicit open‐ended responses. Open questions were employed to encourage participation and to gain a richer understanding of participants’ thinking. All focus groups were audio recorded and transcribed verbatim. One investigator facilitated all focus groups (K.L.) with at least one other investigator present to observe and making field notes (S.B., G.F.C., G.T.H. and N.G.).

**Table 1:** Semistructured interview guide.

Welcome Introduction of facilitators
Background to research
Discussion regarding recording and consent (privacy, confidentiality, reporting findings, storage)
Explain rules of focus group discussion
Questions
*Part 1*
Icebreaker activity
*Part 2*
A diet sheet is generally a paper handout that contains information on the foods you need to eat. What has been your experience with diet sheets? Prompts if needed: did they received one after visiting the dietitian, did you use it during the visit? After the visit. Why/why not?
What were the elements of the diet sheet that you found useful? Prompts if needed: what specific parts were useful to you? e.g., meal plan? Food lists? Explanation why change was needed? Tips on how to change?
What parts of the diet sheet were not useful or you did not like?
Did the diet sheet help you change you diet? Prompts if needed: did you need to look for other information to help you make changes? What kind of information? What things were missing, e.g. food examples that applied to you?
Does anyone have any more to add on the topic of the content of the diet sheets?Prompts if needed: what are your thoughts about things like links to information online? do you prefer positive framed messages? do you want a variety of food examples eg takeaway options, different cultures etc
Part 3—annotating resources
Let's have a look at these diet sheets. I want you to have a look at number 1. What do you like about the content? What do you like about the format? What don't you like? Use the texta to highlight the parts you like and red the parts you don't like
Repeat for all resources
Does anyone have any more to add on the topic of the format of the diet sheets? Prompts if needed: e.g. prefer a small number of pages rather than a book? prefer large font or pictures, how did telehealth impact on this topic
Does anyone have any final comments on how we can design better diet sheets? Would you also like other formats like videos? Podcasts, animations? Anything else?
Finally, lets put it to the vote. When it comes to diet sheets you prefer … (use the discussion to help summarise the key points)
Thank participants and close session

**Table 2:** Summary of resources viewed in focus groups (*n* = 12).

Title	Length (pages)	Hemingway editor readability score	Author
Carbohydrate and Glycaemic Index (GI)^a^	5	7	Baker Heart and Diabetes Institute
How body controls blood sugar levels	1	Not applicable – no words	Illawarra Diabetes Service
10 tips for healthy eating with diabetes^a^	1	9	Diabetes UK
Kidney disease: High‐energy eating	2	5	Nutrition Education Materials Online/Queensland Government
Reading food labels	2	8	Illawarra Shoalhaven Local Health District
Low Tellurium eating plan^b^	4	3	Illawarra Shoalhaven Local Health District
Low Tellurium diet^b^	5	4	Illawarra Shoalhaven Local Health District
Making healthy food choices^a^	6	5	National Diabetes Subscription Scheme
Eating a plant‐based diet with chronic kidney disease	6	10	British Dietetic Association Renal Nutrition Specialist Group/plant based health professionals UK

^a^ Resources listed ere shown to focus group participants as black and white and colour versions.

^b^ The low tellurium eating plan is a copy of the low potassium diet sheets with the element tellurium replacing potassium. The low tellurium diet is a fake diet sheet with elements taken from several common resources under development by local dietitians including use of a clock to remind patients to measure blood glucose levels, food groupings in columns and simplified wording. Pre‐focus group user testing with consumers suggested consumers would feel compelled to follow these unless told specifically not to—so the name of diet was changed from potassium to tellurium at request of consumers in the pilot phase to reflect this.

### 2.3 Analysis

Focus group recordings were uploaded to a transcription software (Otter.ai) which transcribed the audio recordings. The audio recordings were compared against the transcript to ensure the accuracy of the transcriptions. No transcripts were returned to participants for comments, nor were repeat focus groups carried out. Dedoose software [34] was used to manage, store and code data for analysis. This software facilitates coding of transcripts to ensure a transparent audit trail. Thematic analysis followed the approach outlined by Braun and Clark [35]. All transcripts were read multiple times and line by line inductive coding conducted by two people independently. This ensured triangulation of findings and initial categories and themes reflected the range and depth of data collected. Preliminary themes were discussed as a team and final themes derived iteratively by agreement.

Annotations were analysed using manifest and latent content analysis. Manifest coding was completed first, which involved assessing the annotations as they appeared and identifying direct themes based on what was directly written [36]. Following this, latent coding analysis was conducted to understand the consumer perspective more deeply, by coding the meaning behind the annotations. To prevent confirmation bias, latent analysis was conducted after listening to the focus group recording to understand consumer perspectives and the context of the conversation and annotation discussion [36].

## 3 Results

Participant characteristics are shown in Table 3. In total, 45 adults participated in seven focus groups. Participants were predominantly female (73.3%), aged 18–84 years (mean 51.7 ± 23.7) and achieved a high school (37.8%) or university qualification (33.3%). Almost half (42.2%) required glasses to read. While almost half were of Australian born (48.9%), nine other different ethnic backgrounds were represented. There was an even distribution of participants from lower socioeconomic (Deciles 1–3, 24.4%) and least disadvantaged backgrounds (Deciles 8–10, 11.1%). Focus group duration ranged from 70 min to 2 h (mean 97 min).

**Table 3:** Characteristics of participants (*n* = 45).

Characteristics	*n*	%
Gender		
Male	11	24.4
Female	33	73.3
Nonbinary	1	2.2
Age (years)		
18–30	14	31.1
31–40	2	4.4
41–50	2	4.4
51–60	6	13.3
61–70	3	6.7
71–80	14	31.1
81–100	2	4.4
Not reported	2	4.4
Educational attainment		
Primary school	1	2.2
High school	17	37.8
Trade qualification	9	20.0
University qualification	15	33.3
Not reported	3	6.7
Ethnicity^a^		
Australian	22	48.9
Aboriginal Australian	1	2.2
Pasifika	1	2.2
North West European	2	4.4
Southern European	12	26.7
North African and Mid‐Eastern	0	0
Southeast Asian	0	0
Northeast Asian	0	0
Southern and Central Asian	0	0
People of the Americas	1	2.2
Sub‐Saharan African	1	2.2
Not reported	3	6.7
Glasses required		
Yes	24	42.2
No	19	53.3
Not reported	2	4.4
Socioeconomic status^b^		
Decile 1 (most disadvantaged)	9	20.0
Decile 2	2	4.4
Decile 3	0	0
Decile 4	1	2.2
Decile 5	4	8.9
Decile 6	9	20.0
Decile 7	8	17.8
Decile 8	1	2.2
Decile 9	4	2.2
Decile 10 (least disadvantaged)	3	6.7
Not reported	4	8.9

^a^ Ethnicity reported according to Australian Bureau of Statistics Standard Classification of Cultural and Ethnic Groups. Data source: Australian Bureau of Statistics (2019) Australian Bureau of Statistics Standard Classification of Cultural and Ethnic Groups, Australia. Cat no. 1249.0. https://www.abs.gov.au/statistics/classifications/australian-standard-classification-cultural-and-ethnic-groups-ascceg/latest-release#data-downloads.

^b^ Socioeconomic status determined by postcode to determine Index of Relative Socioeconomic Disadvantage. Low score indicates greater disadvantage; high score indicates relative lack of disadvantage. Data source: Australian Bureau of Statistics (2021) Socio‐Economic Indexes for Areas (SEIFA), Australia. https://www.abs.gov.au/statistics/people/people-and-communities/socioeconomic-indexes-areas-seifa-australia/latest-release.

**Figure 1:**
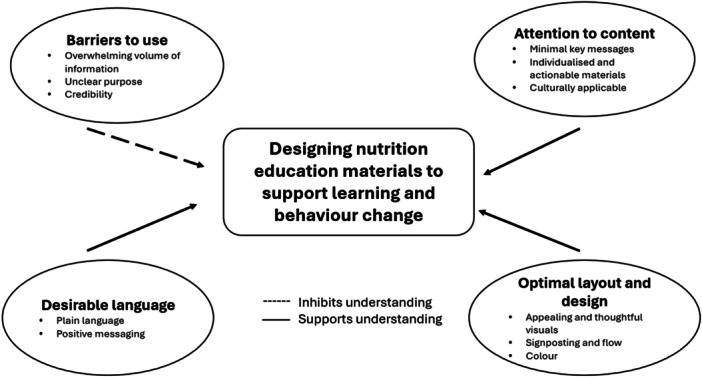
Conceptual diagram illustrating relationship between themes and subthemes.

From focus group discussions and annotations, we identified four recurring themes: barriers to use (overwhelming volume of information, unclear purpose, credibility), desirable language (plain language, positive messaging), attention to content (minimal key messages, individualised and actionable materials, culturally applicable), optimal layout and design (appealing and thoughtful visuals, signposting and flow, colour). Table 4 shows selected quotations to illustrate key concepts in support of each theme or subtheme. Figure 1 depicts the relationships between concepts identified.

**Table 4:** Exemplar quotations to support thematic analysis.

Theme	Subtheme	Illustrative quotes
Barriers to use	Overwhelming volume of information	‘Sometimes these leaflets, they tend to almost overload you with too much information. And so you get bogged down, and you think, I can't be bothered to go any further. You push it to one side and give up.’ ‘Too busy with lots of tables and squares everywhere’ ‘(D'iet sheets) can't be too busy with overwhelming pictures, text and colours’ ‘Very overwhelming sheet with lots of information’ ‘I'm a little bit overwhelmed by how much information there is.’ ‘They gave me all this pile of sheets. And I thought, I got home and I was totally bamboozled. And I thought, yeah, I'm just going to carry on with my diet’
	Unclear purpose	‘The information does not tell you what happens, is it a good thing or a bad thing? I don't know’ ‘Like this (it)…doesn't properly explain to you what exactly they're talking about’
	Credibility	‘Another little pet peeve I had is that this image is clearly just a screenshot of something… It's pixely.’ ‘And it says to me, it's not very official’ ‘I liked it when they had references to go to another website to learn more. Because you know that it's going to be a legitimate dietitian‐approved website. Because people can just Google things. Go down a wormhole, which is a bit dangerous’. ‘Or maybe like the ones that say, if you have any questions, you can contact your dietitian, and they have the dietitians name, mobile number and email, allowing for more open communication’.
Desirable language	Plain language	‘Says limit, but what is the limit? Not detailed enough’ ‘What is a mineral? Is that salt?’ ‘Drink alcohol sensibly? What is a sensible amount?’ ‘Don't make sheets too complicated ‐ keep it simple’ ‘The text of information just seems like the audience is other health professionals’ ‘I was gonna say the summary as well like it's two points and then it says only eat small portions but what are small portions and then on the front page It says be physically active most days. What?’ ‘It's gotta give like amounts yeah.’ ‘I did notice like there's a few bigger words that I feel like not many people would know and there's not really definitions to go with them’ ‘…it's a minefield, I think you've got to have a science degree to understand this and all these?’
	Positive messaging	‘ (The) “heart attack” message is scary’ ‘I like how it's saying choose this choose that. I have a lot of questions about what they've said because it's so unspecific. I don't understand, like, you could interpret it a million ways.’ ‘We've found what the problem is, so let's get you back on track’ … is a much more positive message as to, we don't want you to eat that, and don't eat that’. ‘They go between glucose and blood sugar. So first it says food is converted into glucose then the next one's blood sugar and then it goes to glucose… Yeah so it could get confusing.’
Attention to content	Minimal key messages	‘So this is really simple and easy and makes sense. Like it's not got all the medical terminology and words and stuff.’ ‘It's just too much information crammed on each page.’ ‘Once you start going over multiple pages, your ability to consolidate all that information in your head is not going to be anywhere as near as good if you were doing it on the one page.’ ‘I mean the messages… there are lots of messages.’
	Individualised and actionable materials	‘And then I really liked when they had an example meal plan. The way they can like use that information practically.’ ‘If they had someone help understand each section this would be beneficial…it does not stand alone’ ‘I don't know what action to take from this sheet’ ‘I like the meal plan at the back. I think that's really good that they've put that in. Doesn't make you feel so overwhelmed by all the information.’ ‘I don't mean to be funny, but the amounts are so small and unrealistic. People don't stick to it. If you would a only had half a cup of muesli or oats, you would be starving.’ ‘It needs to give options rather than telling you what to do’ ‘It's not practical yeah… And then we tend to not follow it then’ ‘I like these. You have space to kind of like do your own note taking … on what else you need to do to make it more for you rather than just something everyone's getting.’ ‘I have more than one condition and you've only catered to one… So what do I do? Which one do I follow?’ ‘I'm not a numbers person, you know, that I'm tangible. I have to see. But they told me that you have to have half a cup of pasta. I'm gonna eat that much just tasting if it's cooked, you know’
	Culturally applicable	‘Sometimes when you translate it, it does not make sense’ ‘Haven't seen any information in our own language’ ‘Can the number on the sheet provide me with an interpreter’ ‘Those who come from a different cultural background (there is) no information on where to go to get information in their language’ ‘I think food is not singular items. Food is wrapped up with cuisine, with knowledge… ‘In terms of culturally… So adding one or two things doesn't make it (culturally sensitive) … I know we have incorporated it into our diets, but it doesn't make it culturally sensitive.’
Optimal layout and design	Appealing and thoughtful visuals	‘You can't read the graph in black and white, the lines look the same’ ‘Pictures are good, I would be inclined not to read it if there were no pictures’ ‘I like it because it is large and I can read it’ ‘It's got like, tips to get the right balance of healthy fats. And then in the column right next to it is just like someone chopping vegetables on the chopping board. Yeah it's like the images don't provide really anything extra.’ ‘(photos of real food packages) make it a bit easier if you're going to a supermarket and you can visually recognize the packaging’.
	Signposting and flow	‘I kind of liked how at the start it just kind of told you what exactly the whole thing was about instead of you having to read through it all and then figure it out by myself at the end’. ‘First of all, it takes me a second to get orientated there's no like here's where you start like… I'm like this is really not ideal’. ‘Table extends over two pages and is very disjointed’ ‘Documents with white space, columns and dot points are good’ ‘When everything is in bold make it unclear on what is the important/key information’ ‘I like the layout of the questions as subheadings and the answer directly below it’ ‘If it was across the page, and it just went down in like a chronological order page by page It's easier’. ‘There's not even like a path for your eyes to follow, you just like everywhere.’ ‘If you're lazy, and you're just like skimming it, I could read the headings and pretty much have most of the information I need.’
	Colour	‘I am looking at this sheet and thinking, it would be great in colour’ ‘Colour is appealing you want to read it’ ‘I feel like use the colours to explain not to make it look pretty’ ‘Yeah, you can do like single colours like red for bad, green for good… and just for pictures like that would be enough. Rather than just having black or white or overloading’ ‘What if you couldn't print it out in colour? That would be awful wouldn't it?’ ‘(Colour is better) because it could mean the difference between like an egg or a potato’.

### 3.1 Barriers to Use

#### 3.1.1 Overwhelming Volume of Information

Participants perceived most nutrition education materials provided to them by dietitians or shown in the focus group to contain excessive information. They also spoke of the difficulty this posed when trying to determine the key concepts: ‘too much information is hard to take in’. Lengthy documents added to information overload: ‘once you start going over multiple pages, your ability to consolidate all that information in your head is not going to be anywhere as near as good if you were doing it on the one page’. Participants noted the overwhelming volume of information made most materials unsuitable as standalone items to refer back to and needed dietitian direction to determine key messages: ‘I think (diet sheets) need a verbal explanation from like a dietitian as well …the sheet is not enough. I think it always needs an explanation, and it needs somebody talking them through it as well’.

#### 3.1.2 Unclear Purpose

Participants highlighted that many materials did not make it clear who the intended audience for the material was. Some said an introductory sentence would ‘signpost’ to the reader who should read it and why: ‘It needs a brief on every material…I want a bit more of an intro …like what is it? Why am I like looking at it? Why is it important?’. Others highlighted that there was a high level of assumed knowledge included in many materials which made it difficult for readers to ascertain the purpose ‘this one about GI – I assume it is for diabetics but it doesn't say that for sure so I don't really know for certain’ and another ‘it said to avoid constipation…well that is easier said than done. Sorry, I don't know what I'm doing’. A recurring theme from annotations was an unclear purpose of the sheet noting materials needed a clear statement of purpose ‘I wrote on mine (annotation), … what's this designed to do? Because that one doesn't actually, it just launches you sort of into it’.

#### 3.1.3 Credibility

Materials with clear information about the author and currency of materials were articulated by participants as essential for establishing a sense of credibility and trustworthiness. Some participants described that the absence of author information reduced trust in the material and led to them researching online to ‘double check’ with other sources: ‘like I don't really know if this was written by an expert so I would check up and do my own research again to be sure’. Professional design with aesthetic appeal also enhanced trust in the content of material. Features such as a contact email or phone number/helpline as well as signposting to further information were considered valuable: ‘a link to a website and where to go for more makes the sheet more trustworthy’.

### 3.2 Desirable Language

#### 3.2.1 Plain Language

A desire for materials to be written in plain language was a dominant theme in all focus groups and in annotations. Words such as triglycerides, millimoles, minerals, glycaemic index and carbohydrate were identified as requiring assumed knowledge. An explanation in plain language of medical or nutritional terminology was stated as useful by all consumers. For example: ‘what's a …I don't know what a milli mole is. And it doesn't explain, I was looking for somewhere like description’… ‘Make sure to use simple kind of plain language that's easy to understand’. Other words identified as problematic were common dietetic phrases such as moderation, healthy carbohydrates, healthier fats, carbohydrate counting, intake, limit, energy and protein foods. Consumers expressed strong opinions that these phrases required further explanation. For example: ‘Drink alcohol sensibly? What is a sensible amount?’ and another ‘Don't make assumptions that people know what carbohydrates and proteins mean, explain it’.

#### 3.2.2 Positive Messaging

Many resources described the consequences of dietary non‐adherence. For example, resources relating to the low potassium diet described the cardiac consequences of hyperkalemia, and the diabetes resources outlined the implications of hyperglycaemia. Consumers strongly disliked language that was frightening, judgemental or dichotomous in nature. For example, when describing the implications of high cholesterol ‘If you said it in like a way when someone talks to you, like explaining it…like this is like a low possibility but this is what could happen not like you're gonna have a heart attack and stroke you die’. Another example of an aversion to the language around risk or consequences from one consumer indicated ‘you want it to be a bit softer…and in kind of a conversational style’.

Positively framed pragmatic and realistic messages were also strongly desired. For example, phrases such as ‘avoid' were universally disliked: ‘(I prefer) using terms like cut down slowly instead of avoid… as avoid is unrealistic’ and considered unrealistic in the context of people's lives. For example: ‘So (rather than avoid)…it should tell you more like what you can eat rather than like all the things you should avoid’. Descriptions of food choices were more desirable if written in the format of food swaps*:* ‘So I really think (instead of avoid) try, like swap this for this or increase this’.

### 3.3 Attention to Content

#### 3.3.1 Minimal Key Messages

Consumers indicated that most materials viewed contained too many concepts, and indicated they would prefer materials with a limited number of key messages. In addition, consumers emphasised the importance of explaining key concepts and avoiding assumed knowledge to limit the number of key messages. For example: ‘what are healthy carbohydrates? Tell me, I don't know’ and another explained: ‘Don't make assumptions that people know what carbohydrates and proteins mean… explain it’. Some consumers highlighted that the provision of specific food examples enhanced understanding and application of key messages: ‘(this) has examples of suitable snacks, in detail and this is helpful’.

A number of consumers indicated that they had used strategies to simplify nutrition education material information for their family members: ‘When I receive information for my parents I need to summarise in 3 sentences’. Another indicated: ‘if it (the nutrition education material) is for my parents I will create a simplified version’. This activity of simplifying materials was described as critical when resources did not acknowledge the co‐occurrence of other chronic conditions and family members had multiple comorbidities such as diabetes, high cholesterol, heart failure and kidney disease.

When asked to summarise their preferences at the end of each focus group, there was consensus from all groups that while shorter resources were preferred, they did not mind longer resources as long as they were well designed (using plain language, with a limited number of key messages, and optimal visual design).

#### 3.3.2 Individualised and Actionable Materials

Consumers held very strong opinions about their desire to receive nutrition education resources that could be individualised. This included design elements such as space to make notes (‘I like these (resources)… You have space to kind of like do your own note taking that …on what else you need to do to make it more for you rather than just something everyone's getting’) to more extensive food lists and meal plans that can be personalised (‘my problem with half of these foods are is that I won't eat them…so it's not applicable individualised to you…are they thinking about the patients when they hand this information out?’. The use of second person language (using the active voice) was noted to create a more intimate and engaging experience for the consumer: ‘I liked how they said “you“ and “I”. It felt really personal’.

The other major finding discussed in all focus groups was recognition that many resources were not actionable (‘They're telling you what to do but they haven't given you tools’). Consumers identified that materials did not contain clear explicit instructions which was often related to vague terminology (‘they say have three meals but what is like the right size of these meals? There's not really much information to follow’ and similarly ‘Tells you to choose healthier fats, but what are healthier fats? I don't know what action to take from this sheet’). Desirable features to improve actionability included meal and food examples (‘I like how they have given…practical ideas like low GI carbohydrate foods like pasta's, low GI rice and now I'm like, oh okay, these are some examples’), explanations of key concepts (‘I like this, it's explained …because if some people were to read triglycerides, like what the hell is that but at least it says that means fat, so that's good’) and use of conversational language (‘I like the layout of the questions as subheadings and the answer directly below it’).

#### 3.3.3 Culturally Appropriate

Meal food lists were considered highly desirable by all consumers. Numerous consumers discussed the challenges posed when foods featured in lists were not culturally appropriate or served in a manner unfamiliar to that culture. For example*: ‘*they told me that you have to have half a cup of pasta. I'm gonna eat that much just tasting if it's cooked…So like, it's (important) how it fits into my (Italian culture) world …I need it very practical’. Annotations on the materials suggested the preferred layout of food lists were those which included a diverse range of foods from many cultures.

### 3.4 Optimal Layout and Design

#### 3.4.1 Appealing and Thoughtful 3 Visuals

Consumers expressed a strong desire for informative rather than decorative visuals, diagrams and graphs. In addition to visual appeal (‘I would be inclined not to read it if there were no pictures’) images serve to entice the reader and enhance understanding. This is particularly useful in those with potentially weaker reading skills: ‘Yeah, those (images) are … what I can eat, those are good options, rather than having to read it’. Salient features of visuals that consumers identified as important were location (including visuals alongside the relevant text) and a figure legend (to highlight the purpose of the image). Consumers also preferred images of foods that were specific, explicit and included brand names rather than generic images of food products. This was perceived to assist with location in the supermarket when shopping. Images of people or foods relevant to the target audience were also considered important to consumers. A variety of food examples relevant to many cultures was also emphasised.

Of note and highlighted by many consumers were usual of simplified visuals of organs or glasses. Supporting Information S1: Table 2 highlights the issues consumers faced with identifying the stomach on a diabetes resource and in interpreting the correct volume of a standard alcoholic drink when block colours were not used thoughtfully.

#### 3.4.2 Signposting and Flow

Consumers perceived the visual design of materials as critical to facilitating understanding. Features such as appropriate font size, logical layout, use of images and colour, and formatting were highlighted. The use of columns was controversial, with older consumers suggesting this style reminded them of newspaper layouts, and younger consumers disliked this approach: ‘like you're saying one thing on the left‐hand side and another thing on the right‐hand side. And you kind of reading down the page in a narrow column where if it was across the page, and it just went down in like a chronological order page by page it's easier’.

Signposting of information was considered important. This included an initial paragraph to situtate who the potential audience is, use of questions (signposts) regarding the topic in logical order, highlighting, sectioning or bolded points for important information. Use of headers was important (‘a summary is not required (here) because there are headers for different sections’). Consumers also preferred a predictable format with too many features considered a distraction: ‘There's not even like a path for your eyes to follow, you just like (look) everywhere’.

#### 3.4.3 Colour

Resources in colour were considered superior and more appealing than black and white versions: ‘We all picked up the coloured ones… The pictures and everything as well, like they just, they stick out… And then you.go like, Oh, this looks delicious. Maybe I do want to eat healthier’. However, consumers indicated that use of colour needs careful consideration in addition to other visual design elements. For example: ‘I think less colours would be better. Yes. Too many colours. Yeah and has it been tested for people with colour blindness?’. Consumers indicated that materials should be designed from the outset with the printing format in mind: ‘They need a produced one that is black and white printable’.

Design elements considered important to consumers alongside colour were maximal use of white space to avoid the feeling of information overload (‘(information) could be like spread out…separated a bit…because everything's just shoved so close together, it's a bit much’), careful use of bolding to emphasise key points only (‘when everything is in bold it makes it unclear on what is the important/key information’) and use of bullet points.

Using these findings and relevant literature on universal design and evaluation of patient education materials [9, 11, 17, 37, 38, 39, 40, 41, 42, 43, 44, 45, 46, 47, 48, 49, 50], a framework for dietitians has been developed. This is shown in Table 5. This can be used when developing new materials or to evaluate existing ones. Further guidance and an exemplar resource template is published elsewhere [17].

**Table 5:** Framework for evaluation and development of nutrition education materials.

PURPOSE □ Is the intended purpose of the material clear? □ Does the material explain the reason for the main messages? □ Are signposts provided to find further information (e.g., weblinks, QR code)? ORGANISATION □ Is the main message at the top/beginning/front of the material? □ Does the material present information in a logical order or sequence? □ Does the material provide a summary of main messages/key points? □ Do tables and figures have titles and headings where needed? □ Is the material accessible to those with disabilities (e.g., consider use of screen readers, colour blindness)? □ Is left alignment used (full justification can add spaces that distract readers)? CONTENT □ Is the material written using the active voice and addressing the user directly? □ Is the material written in plain language? □ Does the material minimise ‘dietitian speak’ (e.g., intake, energy, moderate)? □ Are acronyms, symbols, and medical or dietetic terms explained? □ Does the material use syntactical strategies to reduce cognitive demand (e.g., short sentences ≤ 25 words)? □ Is the material written at an appropriate grade level (ideal primary school level)? □ Is there at least one action outlined that a reader can take to improve their health? □ Does the material give concrete/explicit examples (consider brand names of packaged foods)? □ Does the material consider cultural norms, traditions, beliefs around food and health? □ Are calculations explained and information provided on how to use (e.g., food label reading)? □ Does the material break down actions into manageable steps? □ Does the material use positive messaging? □ Are common household measures or analogies used (e.g., palm sized portion)? DESIGN □ Does the material have a consistent style of presentation (font, layout)? □ Is the font used a sans serif style to improve comprehension? □ Is the font size large enough for the intended audience? □ Is there adequate white space? □ Does the material use signposts in the form of questions for users or informative headers? □ Does the material break/chunk information into short sections? □ Does the material use visual strategies to draw attention to key points (e.g., box, bolding, bullet points)? □ Does the material use strategies to reduce cognitive demand (e.g., bullet points, lists)? □ Does the material use appropriate visuals familiar to the target audience to assist understanding? □ Are visuals of high quality, uncluttered and labelled? □ Are visuals located adjacent to relevant text? □ Is there use of colour to illustrate key points? □ Are the key messages lost if printed in black and white? □ Does the material direct the user to other further information? □ Does the material contain space for user interaction/individualisation (e.g., space for notes)? SOURCE CREDIBILITY □ Is it clear who the author is and when the material was written? EVALUATION □ Obtain feedback from diverse consumers prior to release □ Consider the demographics of the target audience when writing– age, gender, vision, font size, income, ethnicity, emerging cultural groups and nutrition literacy skills □ Ensure diverse food examples are provided when writing for general audiences

## 4 Discussion

It is now an expectation in many developed nations that health professionals, including dietitians engage with consumers regarding the provision of healthcare services [51, 52, 53]. Consumers possess important skills, experience and knowledge that can be used to enhance the quality of dietetic services delivered. Nutrition education education resources are a key area where consumer feedback is highly meaningful and informative. Three key findings of this study were evident regarding consumer perceptions of written nutrition resources. First, current materials exhibit many features that act as barriers to use. Second, both content and design are important features to consumers. Finally, specific features regarding the content are an important area for improvement. This includes minimal key messages, individualised and actionable materials, and culturally applicable materials.

The findings of the present study contribute to the limited evidence base regarding nutrition education material design for dietitians. Three previous studies have used qualitative methods to gain consumer feedback on dietetic resources. Similar to the work of Shepherd et al. [26], Clark‐Mercer et al. [54] and Meyer et al. [29], nutrition education materials were associated with consumer information overload, frustration with the lack of plain language and concerns regarding design features. This is also similar to work in other fields such as oral health [55] and diabetes education [22].

**Figure 2:**
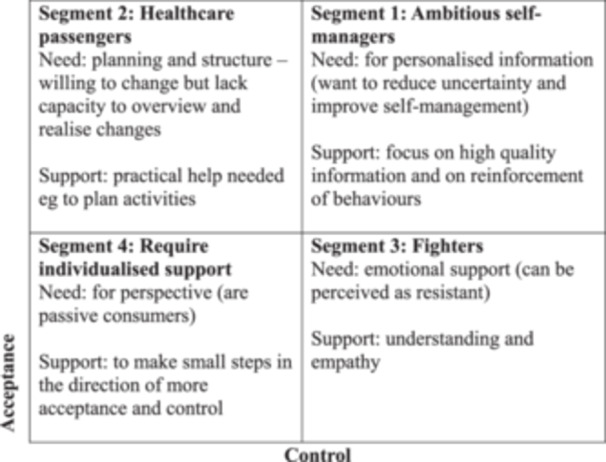
Four segments of healthcare consumers. Adapted from Bloem et al. [56].

Since these early qualitative works in dietetics, there has been important expansions in our understanding of the psychological needs of healthcare consumers [56]. Derived from the field of marketing, ‘psychographic segmentation’ seeks to understand and characterise the drivers of consumer engagement, levels of patient activation, priorities and communication preferences of healthcare consumers [57]. This has been extended to profile consumers who follow healthy eating patterns [58, 59]. The findings suggest there are four segments of healthcare consumers (shown in Figure 2). Work in a large Dutch cohort (*n* = 2465) [56] identified that approximately one‐third of healthcare consumers were placed in either segment 1 (ambitious self‐managers requiring high quality information) or segment 4 (unable to or unwilling to improve their health, requiring individualised advice). The relevance of this work to the present study is clear. Themes regrading a preference for credible high‐quality information and a desire for actionable resources that can be tailored to incorporate individualised advice were dominant in all focus groups.

An important finding in this study was the explicit desire by consumers to include brand names and images of foods in nutrition education materials. This is in contrast to current practice, where many jurisdictions prohibit use of brand names in education materials. This may be related to copyright concerns, concerns regarding changes in ingredient composition, or a desire to avoid stigmatisation of favouritism of certain manufactures/products. This practice is in contrast to research evidence which indicates education materials using images increases attention, comprehension, recall and adherence to instruction [60, 61, 62, 63, 64]. This concept of ‘pictorial superiority’ is nuanced and as suggested by our consumers generic food images may create confusion rather than support understanding [50] and serve as barriers to making appropriate food choices. Feedback from consumers regarding the redesign of the UK EatWell plate is also similar with consumers indicating food photography is easier to recognise and is inspiring, whereas drawn food images were considered childish [65]. Clear images have also been associated with improved dietary knowledge [66], drawing attention and eliciting emotion [67] and could be used by dietitians to increase the appeal of healthy food choices.

Differing preferences for use of columns according to age was an unexpected finding of this research. Older consumers preferred a multiple column ‘newspaper style’ layout and described this format as ‘familiar’. In contrast, younger consumers (< 25 years) preferred a wide format which has been described elsewhere as more familiar to generations accustomed to mobile phone or online scrolling [68]. Singular column style of information layout has been shown to be more effective and result in greater message comprehension [69, 70, 71, 72]. It is also important to note that dietitians must be aware of legal requirements when designing written education materials. For example, in Australian government employees are obliged to make all content accessible to people with a disability according to the Disability Discrimination Act [73]. This means that features such as columns make materials inaccessible for people with screen readers and should be discouraged as they typically read from left to right, top to bottom [74].

Another unexpected finding from this research was the desire to have links to further information signposted. This included use of QR codes which consumers agreed to be a widely accepted method for signposting information due to COVID 19 pandemic experiences [75]. However, widespread inclusion of QR codes may worsen inequity for those who lack access to relevant technologies [76]. Credibility of materials was also enhanced when explicit details were included regarding the author, date of currency and methods to contact for further information. Further research with end users as technology evolves would be useful to inform future design developments.

There are several strengths of this research. These include the rich detailed descriptions of consumer experiences, priorities and preferences of dietary education materials. Insights were also obtained from a range of consumers across varying age, gender and cultural groups. However, wider cultural perspectives are required, including from consumers in which English is not a first language. This was limited by the cost of interpreter services in the present study. Insights from health consumers who may have limited health literacy, multimorbidity or altered cognitive capacity such as those who have a traumatic brain injury or stroke or other types of neurocognitive decline are also needed. The perspectives of children would also be insightful including those with chronic conditions and those transitioning to adult care. Experimental work using eye tracking would also be informative to quantify the manner in which people read dietary materials.

## 5 Conclusion

Designing effective written nutrition education materials is a constantly evolving activity for dietitians. Passive provision of ‘one size fits all’ nutrition education materials is outdated and does not meet consumer expectations. Instead, dietitians should aspire to provide materials that are designed to meet consumer needs and expectations and can be individualised. Technological advances such as use of large language models, graphic design programs for laypeople and an improved understanding of the science of learning will ensure the quality of written information will continue to evolve. The findings from this study and the newly developed framework provide dietitians with practical guidance to design nutrition education materials that meet consumer needs and expectations and may be a useful starting point for dietitians to improve the quality of their resources.

## Author Contributions

Project conceptualisation: K.L., S.B., N.B., N.G. and G.F.C. Data collection: K.L., S.B., N.G., G.T.H. and G.F.C. Data analysis: K.L., S.B., N.B., N.G., G.T.H. and G.F.C. Writing initial draft: K.L., S.B., N.B., N.G., G.T.H. and G.F.C. Drafting final version: K.L., S.B., N.B., N.G., G.T.H. and G.F.C.

## Ethics Statement

Approved by the NSW Health/Illawarra Shoalhaven Local Health District Human Research Ethics Committee (approval number 2021/ETH12201).

## Conflicts of Interest

The authors declare no conflicts of interest.

### Peer Review

The peer review history for this article is available at https://www.webofscience.com/api/gateway/wos/peer-review/10.1111/jhn.70041.

## Supporting information

Supporting information.

## Data Availability

The data that support the findings of this study are available from the corresponding author upon reasonable request.
