# Supplementary material for: Designing Better Resources: Consumer Experiences, Priorities and Preferences Regarding Contemporary Nutrition Education Materials
Source: J Hum Nutr Diet. 2025 Mar 25;38(2):e70041. doi: 10.1111/jhn.70041 (PMC11934846; doi:10.1111/jhn.70041)
Supplement: Supplementary file 1 — Supporting information. [file JHN-38-0-s001.docx]

**List of tables**

Supplementary table 1. Materials provided to consumers for evaluation

Supplementary table 2. Latent and manifest analysis of annotations

**Supplementary table 1. Materials provided to consumers for evaluation (front page)**


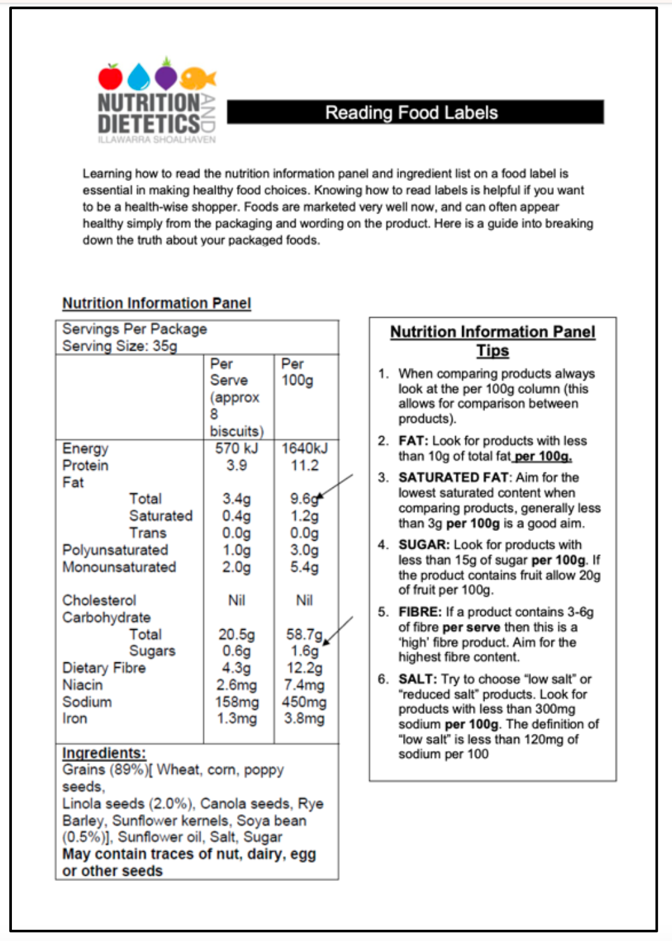

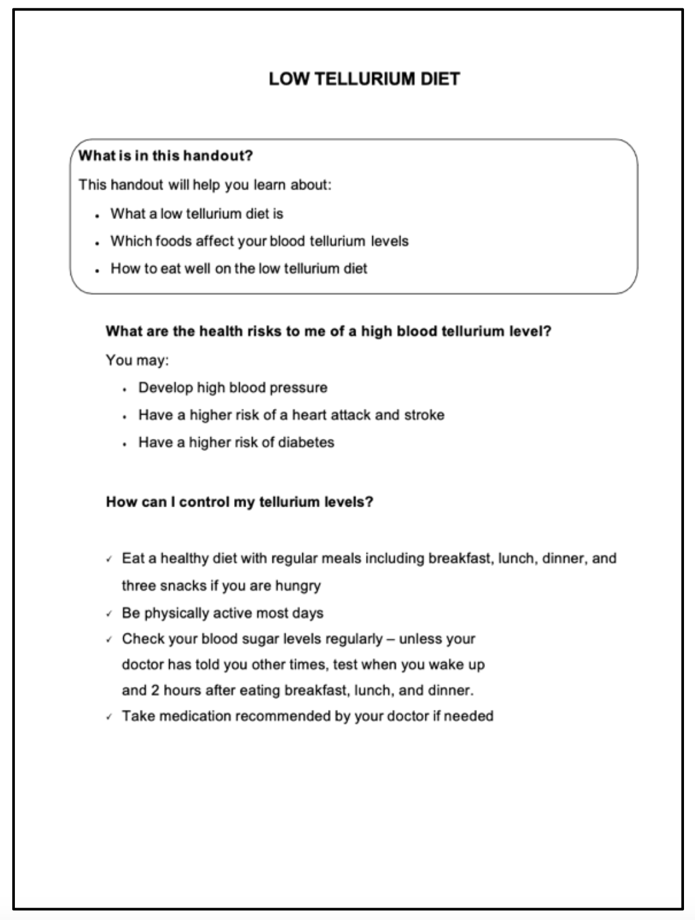

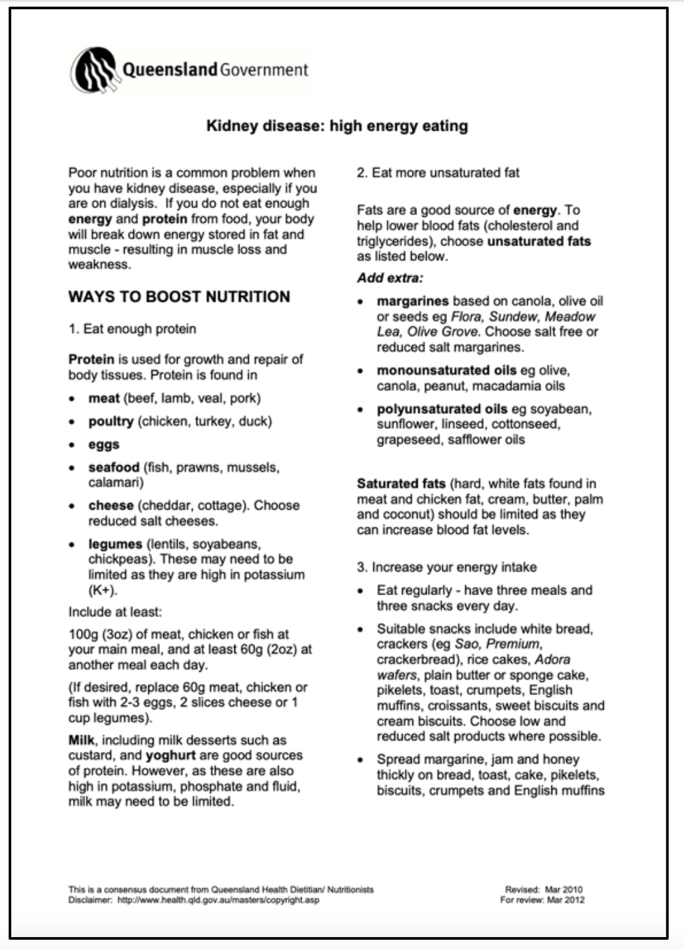

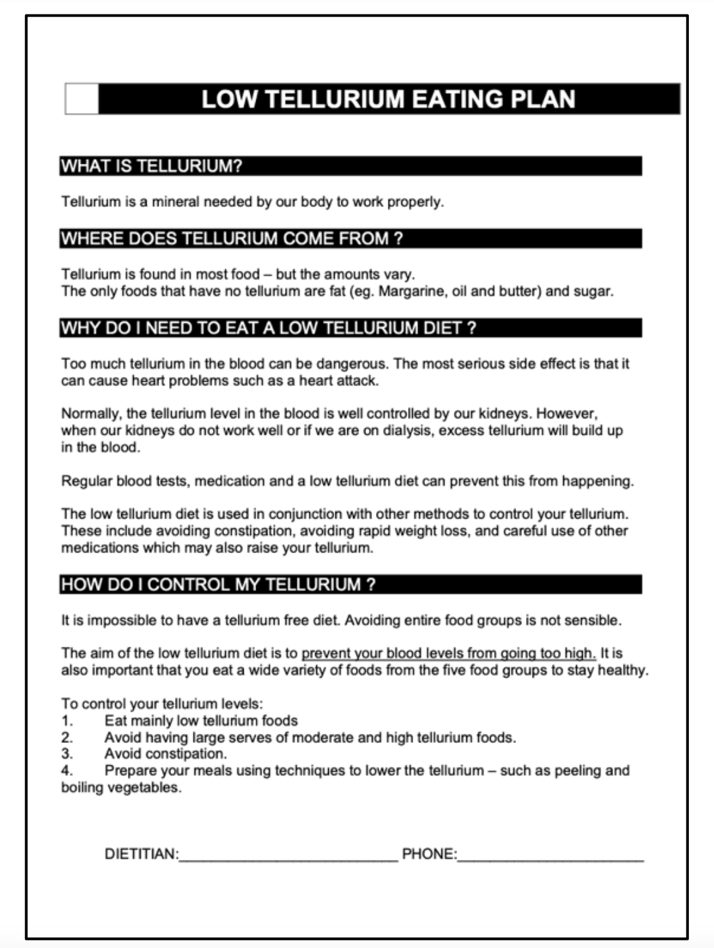


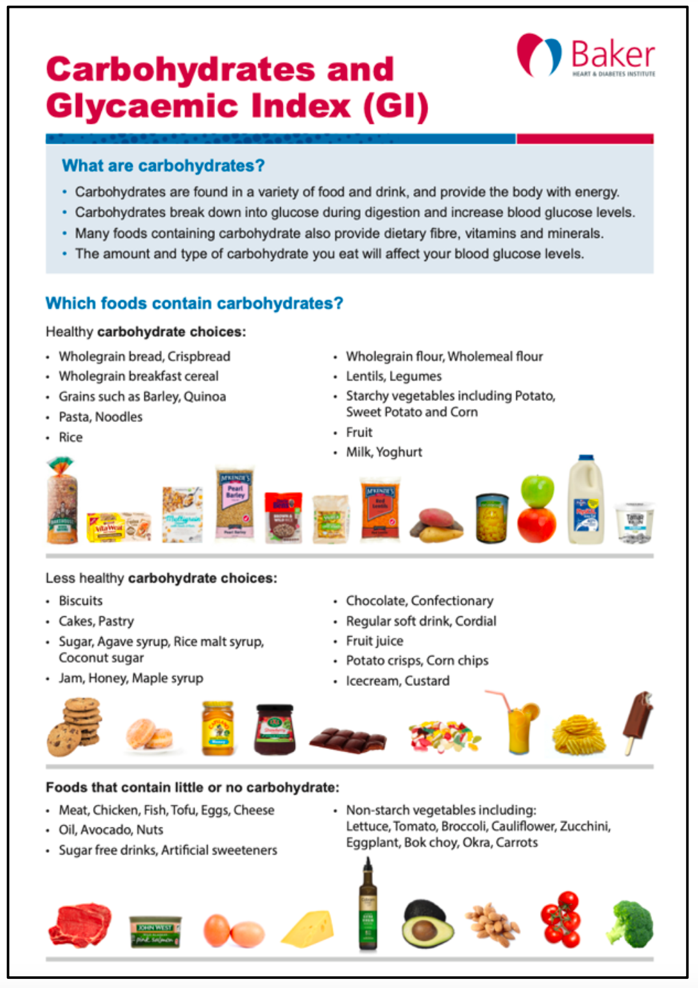


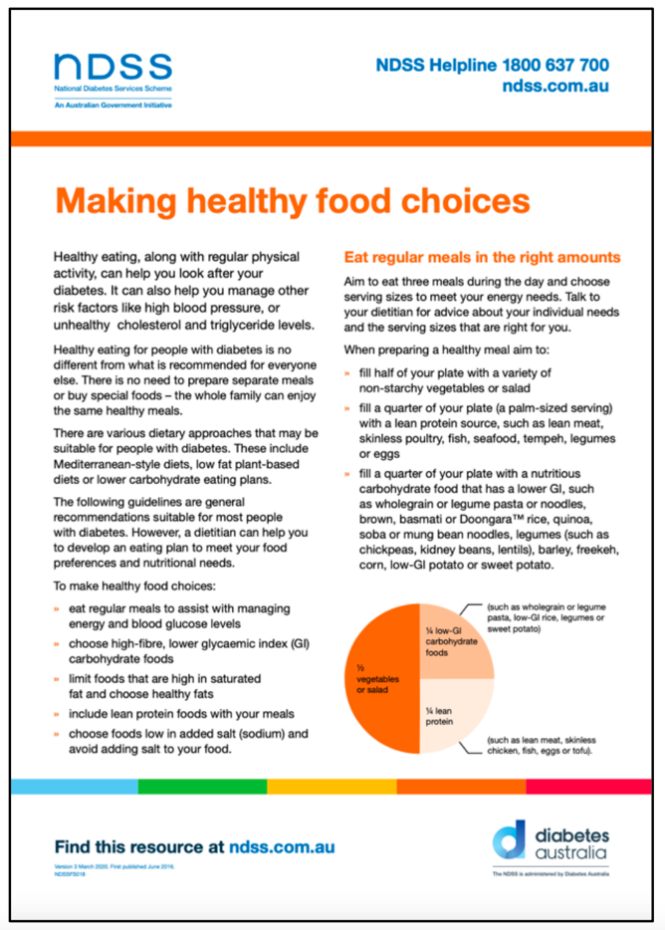

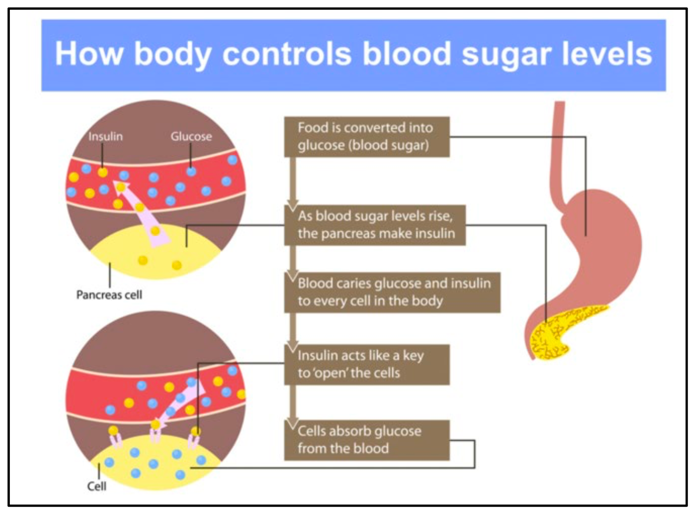

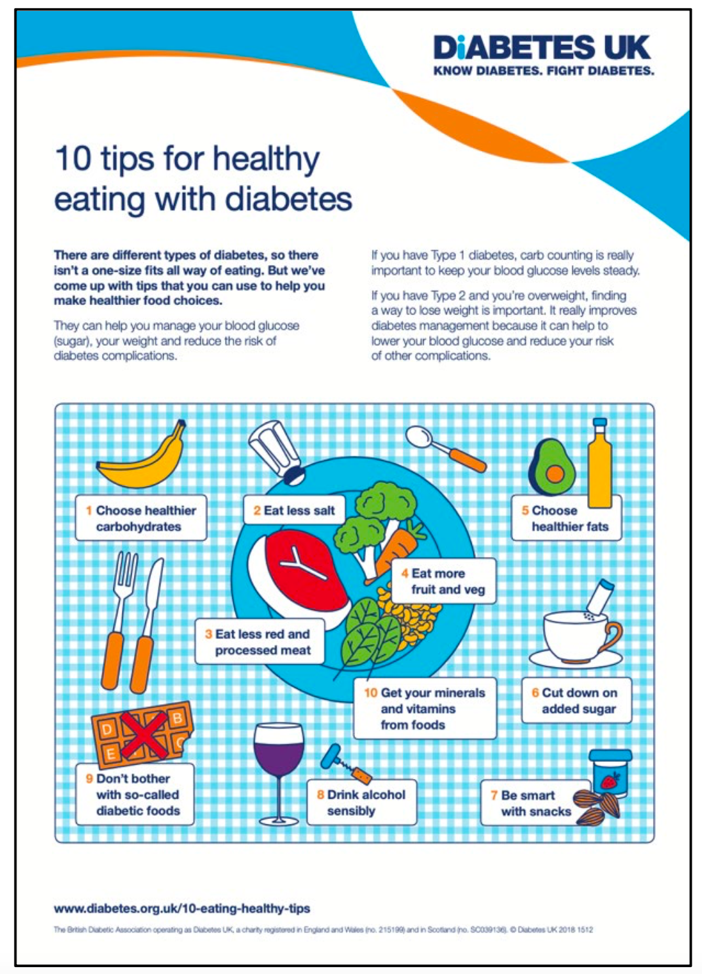


**
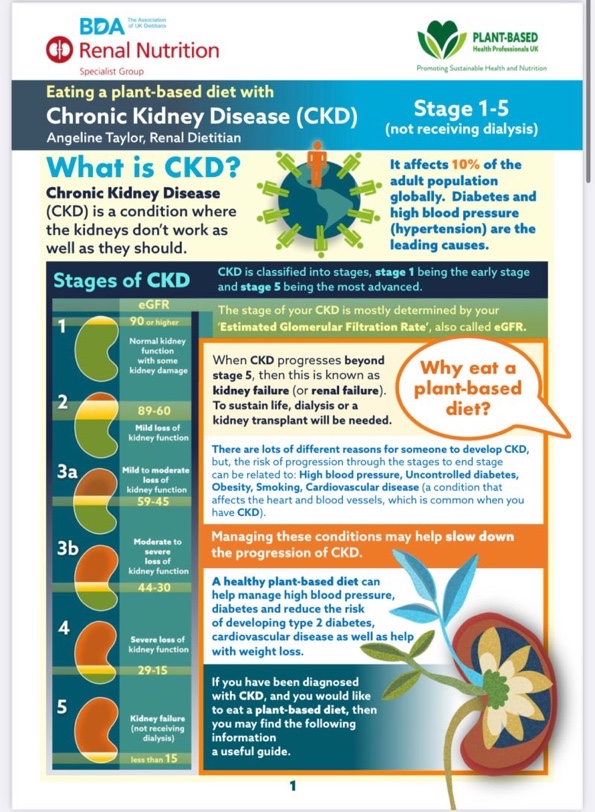
**

**Supplementary table 2. Latent and manifest analysis of annotations**

| **Theme** | **Exemplar annotations** |
| --- | --- |
| **Barriers to use** |  |
| *Overwhelming volume of information* | 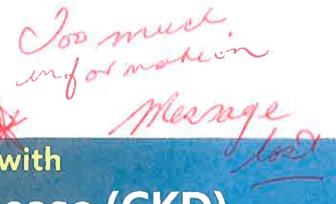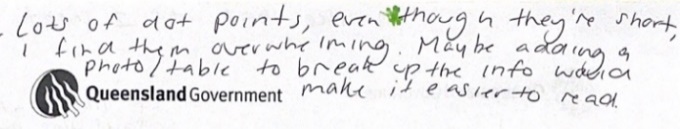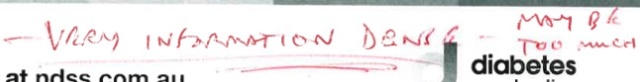**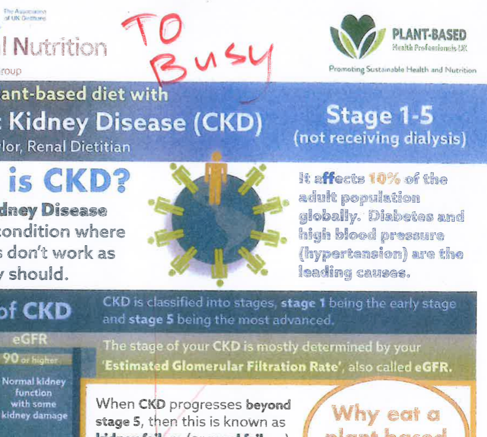 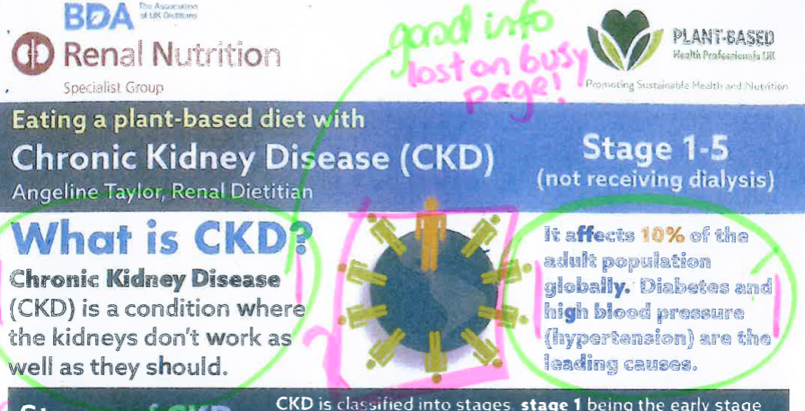**  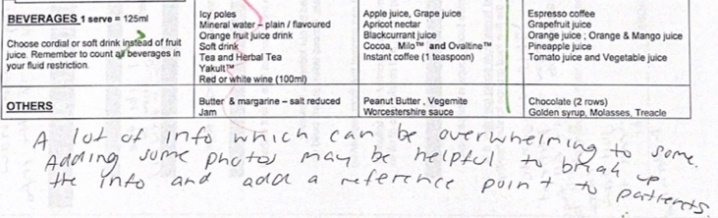 |
| *Unclear purpose* | 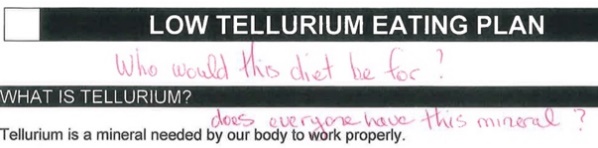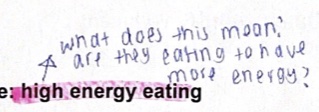**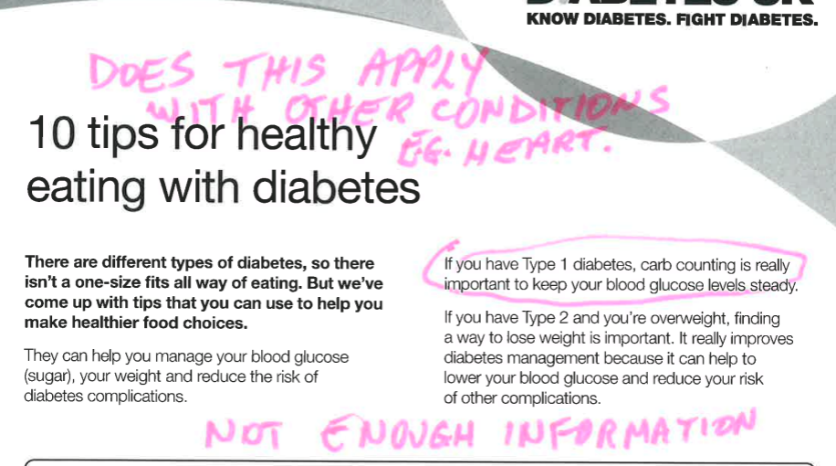** |
| *Credibility* | 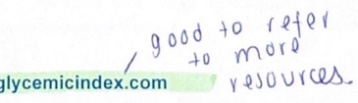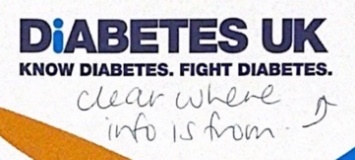  **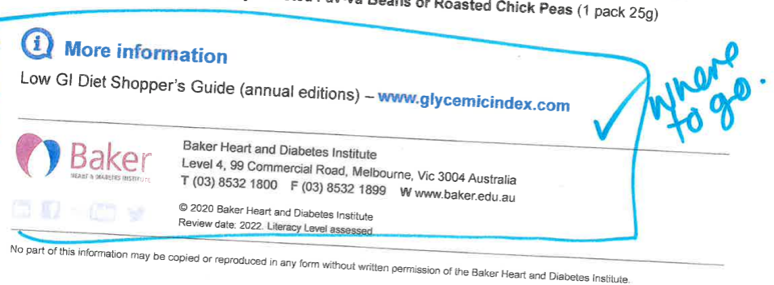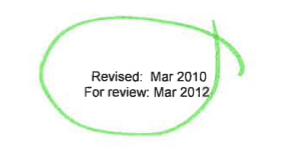**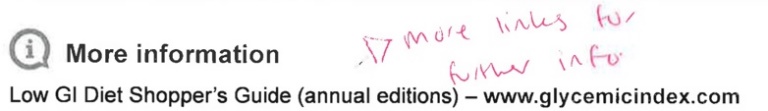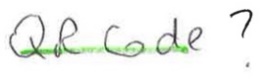**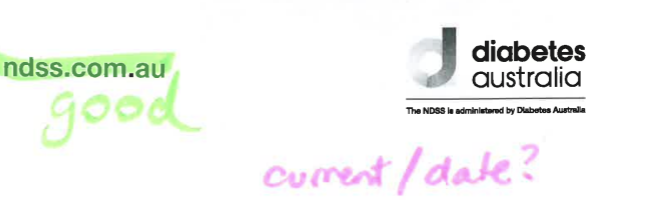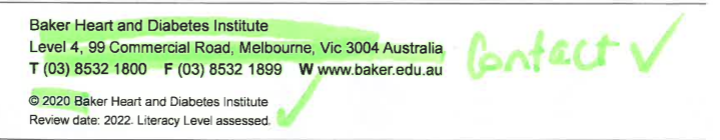** |
| **Desirable language** |  |
| *Plain language* | 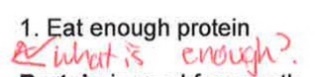**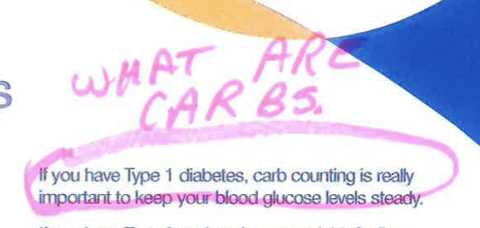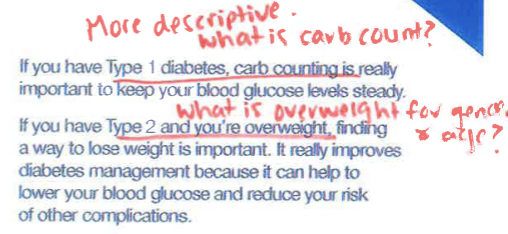** |
| *Positive messaging* | 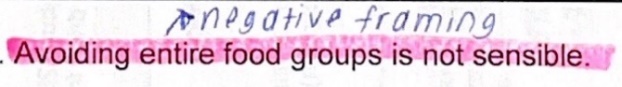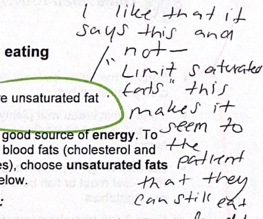*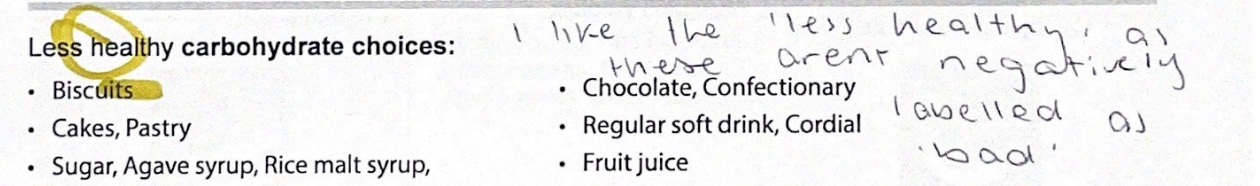*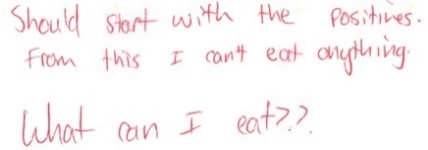 |
| **Attention to content** | |
| *Minimal key messages* | 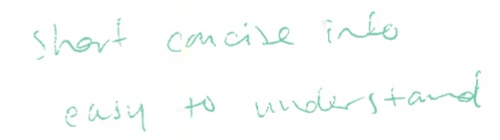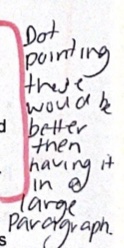 |
| *Individualised and actionable materials* | **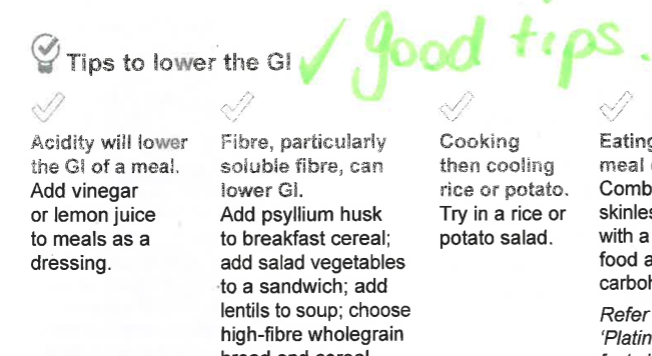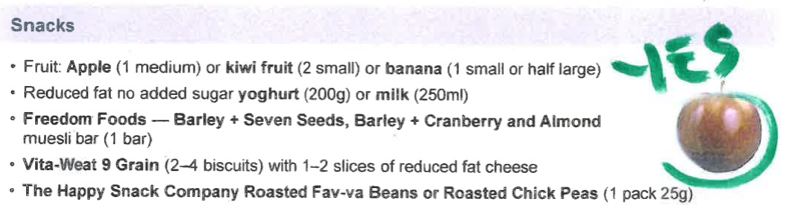**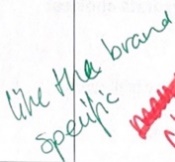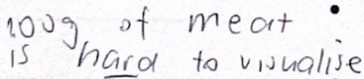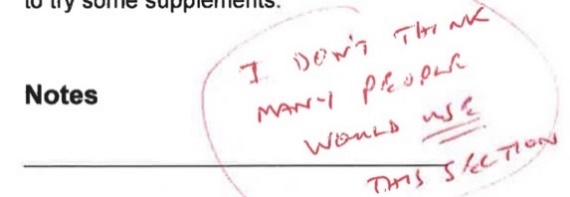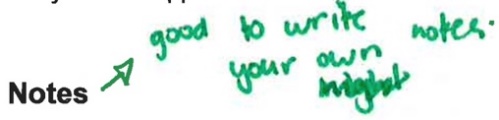**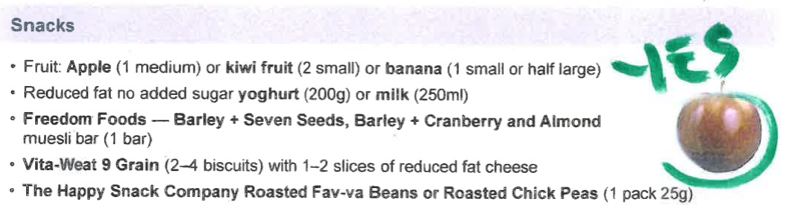 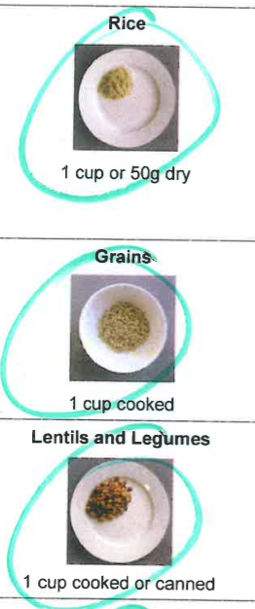** |
| *Culturally applicable* | **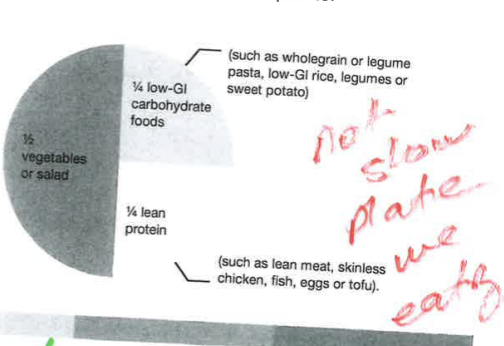**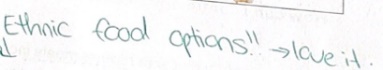 |
| **Optimal layout and design** | |
| *Appealing and thoughtful visuals* | *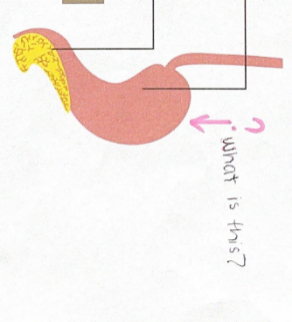*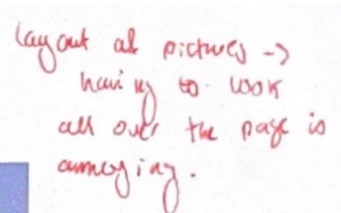**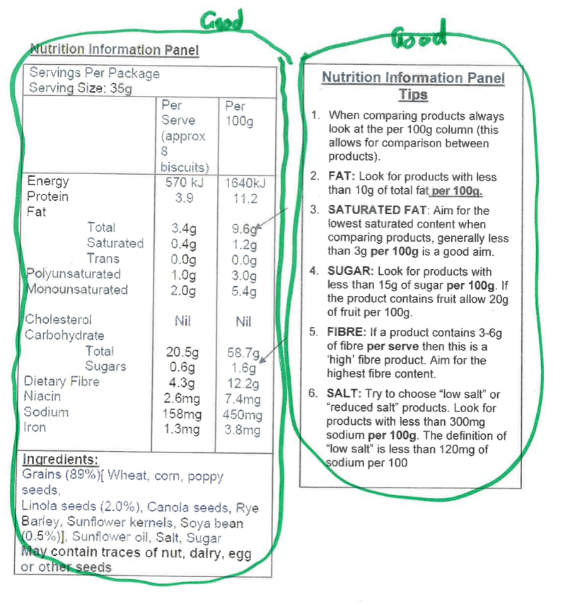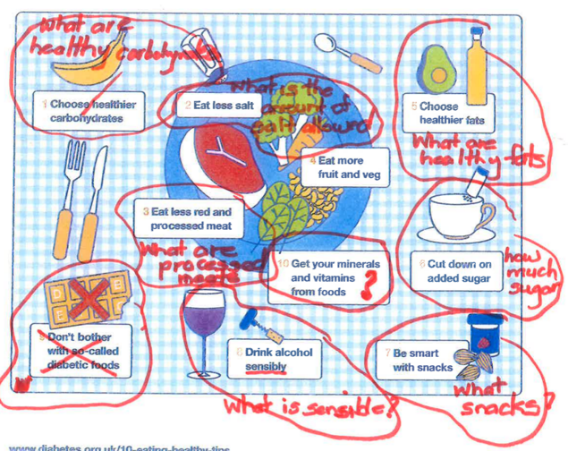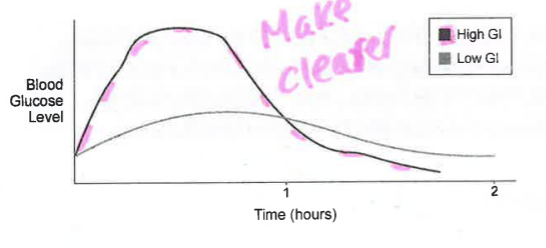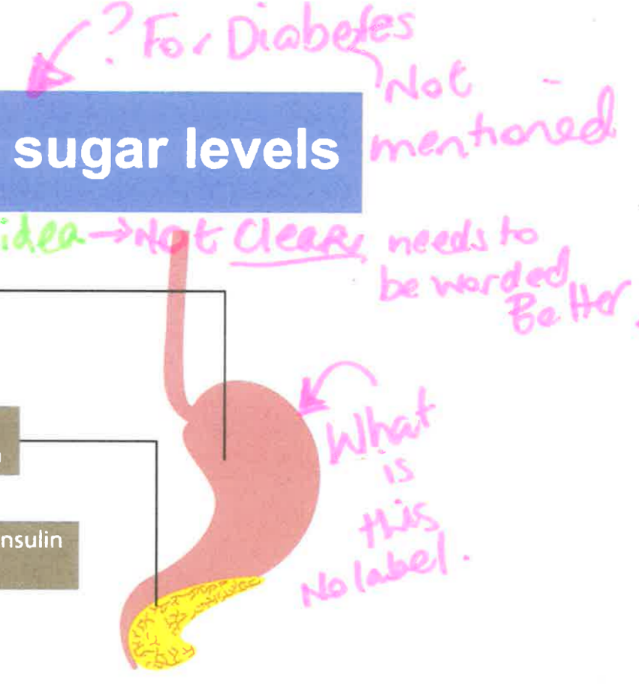**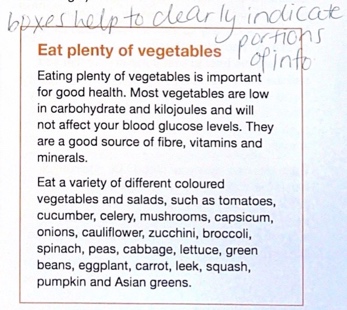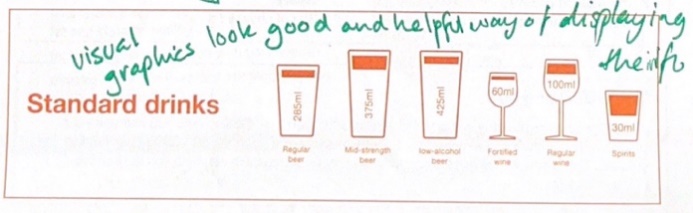 |
| *Signposting and flow* | **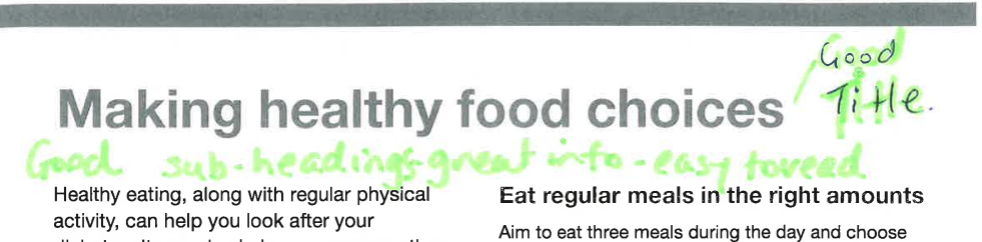**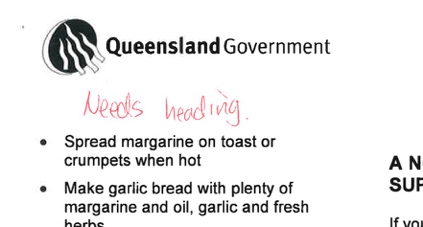  **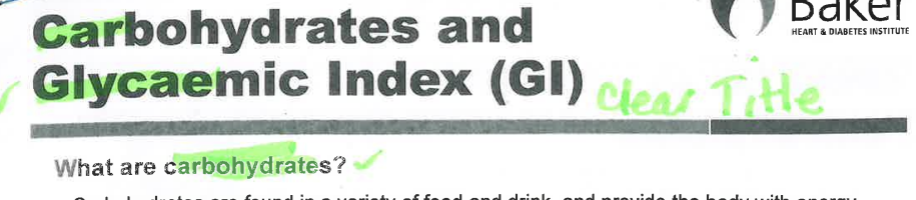**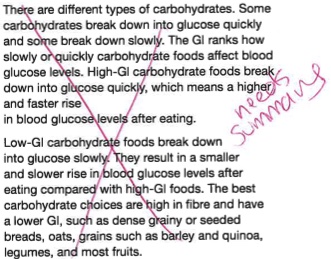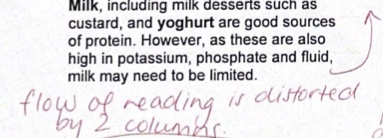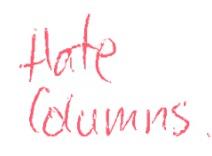  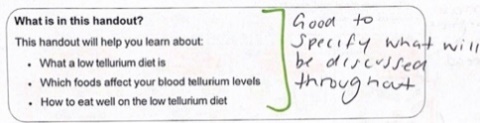  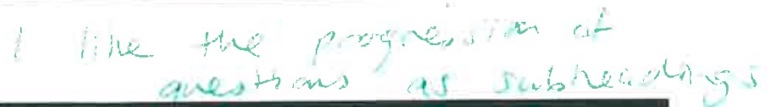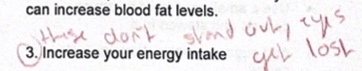 |
| *Colour* | 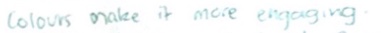**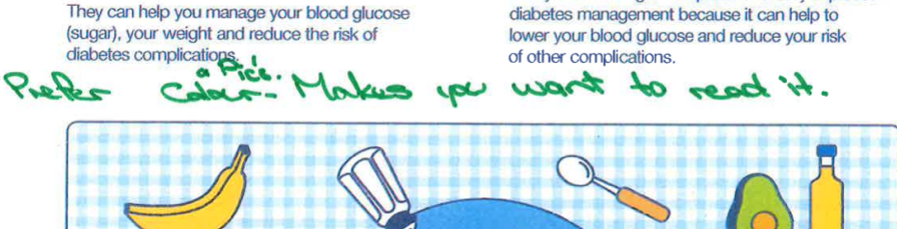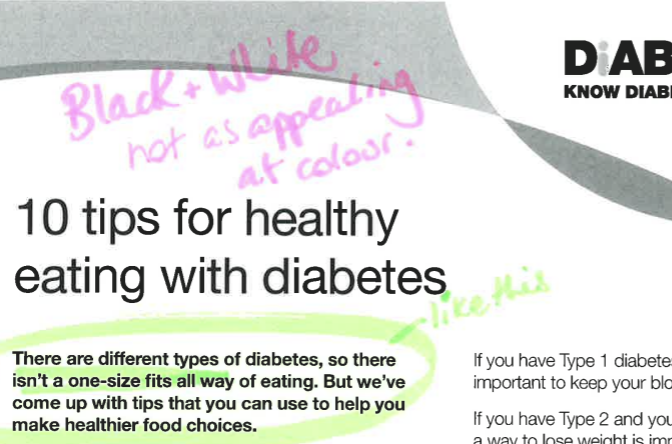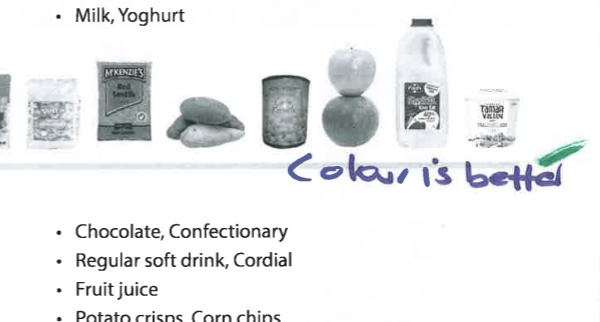** 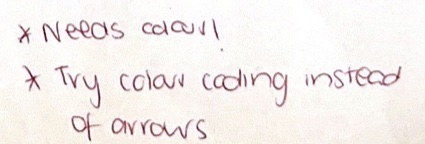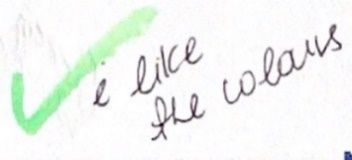 |
